# Genetic relationship in mulberry (*Morus* L.) inferred through PCR–RFLP and *trn*D-*trn*T sequence data of chloroplast DNA

**DOI:** 10.1080/13102818.2014.928980

**Published:** 2014-08-22

**Authors:** Dechang Hu, Ping Zhang, Yan-Lin Sun, Shumin Zhang, Zhaohong Wang, Chuanjie Chen

**Affiliations:** ^a^ College of Life Science, Ludong University, Shandong, Yantai, P. R. China; ^b^ Sericulture Research Institute of Shandong Province, Shandong, Yantai, P. R. China

**Keywords:** *Morus* L., cpDNA, PCR-RFLP, *trn*D-*trn*T sequence, genetic analysis

## Abstract

Ten universal primer pairs of the plant chloroplast genome were used to amplify the chloroplast DNA (cpDNA) non-coding regions in eight mulberry (*Morus* spp.) genotypes, including *M. mongolica*, *M. bombycis*, *M. alba*, *M. atropurpurea* and *M. multicaulis*. Subsequently, the polymerase chain reaction (PCR) products were digested by seven restriction enzymes and the *trn*D-*trn*T fragment for sequence alignment, and the variations were expected to provide the genetic information for system classification. The results from this study showed that: (1) 10 cpDNA primer pairs could be used for successful amplification in the tested materials, with approximately 17.1 kb of the chloroplast genome analysed. The 152 marker loci were detected by 70 primer/restriction endonuclease combinations, among which the *trn*D-*trn*T non-coding region digested by *Alu*I, *Hinf*I, *Mva*I and *Rsa*I was detected by visible fragment length variation in different genotypes of the genus *Morus*. (2) eight *Morus* L. genotypes were divided into two groups based on the digesting pattern discrepancy through cpDNA. The *M. multicaulis* genotypes displayed diversity on an intraspecies level. ‘Nongsang No.12’ was identical with the female parent ‘Beiqu No.1’ (*M. atropurpurea*) in the surveyed sequence, but different from the male parent ‘Tongxiangqing’ (*M. multicaulis*), suggesting that the cpDNA was maternal inheritance in *Morus* L. (3) There were two deletion fragments (451–456 bp; 840–863bp) and six base point mutations in the *trn*D-*trn*T region based on homologous sequence alignment. The sequence of *trn*D-*trn*T in the cpDNA of mulberry could provide more genetic information for phylogenetic analysis and pedigree identification.

## Introduction

Mulberry (genus *Morus*, family Moraceae) is an important economic woody crop for many years because its leaves are the sole food for the domesticated silkworm. It can be propagated both asexually and sexually, and easily hybridized both naturally and artificially. In China, there are mainly 15 species and 4 varieties with about 3000 mulberry germplasm resources, which were widely distributed in different regions. Chromosome numbers of the genus *Morus* are complex, e.g. 2*n* = 2*x* = 28, 2*n* = 3*x* = 42, 2*n* = 6*x* = 84, even 2*n* = 22*x* = 308. These features make the genetic background of *Morus* rather complex. Plant taxonomists generally classify the genus based on the female style, stigma with hairs or protrusions, supplemented by branch, leaf, flower and fruit shape description. However, this method of morphological and phenological identification is difficult, ambiguous, time-consuming and subjective.

With the development of molecular biology, random amplified polymorphic DNA (RAPD),[1,2] amplification fragment length polymorphism (AFLP),[3] inter-simple sequence repeat [4–6] and simple sequence repeat (SSR) [7,8] molecular markers were used in studies on the genetic diversity and phylogeny of mulberry plants. However, the previous results showed that the genetic relationships of mulberry were very complex and the taxonomic status was obscure. Unlike the nuclear genome, the cpDNA has uniparental mode of inheritance and low mutation rate of construction and sequence. The chloroplast could eliminate the interference in identifying the origin and evolution of the allopolyploid genome based on nuclear genome information. It has been considered to be an ideal system in phylogeny and population genetics.[9–11] Sequence comparison or restriction analysis of fragments amplified with universal primers for cpDNA is now widely employed in species identification, genetic diversity and phylogenetic studies in different plant species.[12–14] Some preliminary studies have been conducted on the mulberry chloroplast genome,[15–18] but the genetic information is relatively limited for the systematic classification of the genus *Mo*rus. Chen et al. [15] suggested that *trn*L-F and *rps*16 sequence information sites in *Morus* were of limited value for the purposes of phylogeny. Nepal and Ferguson [16] used sequence data from the internal transcribed spacer (ITS) of nuclear ribosomal DNA (nrDNA) and the chloroplast *trn*L-*trn*F intergenic spacer to study phylogenetic relationships of *Morus*, and the result showed phylogenies based on separate data sets were not statistically significant.

In this study, 10 universal primer pairs of the chloroplast genome were used to amplify 17.1 kb chloroplast DNA (cpDNA) non-coding regions in eight *Morus* genotypes, including five species: *M. mongolica*, *M. bombycis*, *M. atropurpurea*, *M. alba* and *M. multicaulis*. The amplified cpDNA fragments were digested by seven restriction endonucleases. Subsequently, the *trn*D-*trn*T region easy sequenced, and the length polymorphism based on the homologous sequence alignment was detected. The objectives of this research were to evaluate the interspecific and intraspecific chloroplast genome variations based on base mutation or deletion, and also to identify phylogenetic relationships in genus *Morus*. Based on strict maternal genetic characteristics, this result would provide more genetic information for the classification and system evolution of mulberry.

## Materials and methods

### Plant materials

Five *Morus* species including eight genotypes were used in this study. They were sampled in the Sericultural Research Institute of Shandong Province. The ploidy level and origin with collection sites are presented in Table 1. Among the materials, the genotype ‘Nongsang No.12’ (*M. multicaulis*) was the hybrid offspring of ‘Beiqu No.1’ (*M. atropurpurea*) × ‘Tongxiangqing’ (*M. multicaulis*). ‘Xinyizhilai’ (*M. alba*) was the progenitor of ‘Yizhilai’ (*M. alba*) × ‘Guosang No.21’ (*M. alba*), and ‘Fu No.1’ was obtained by radiation breeding from ‘Yizhilai’ (*M. alba*).

**Table 1.** Materials used in this study.

No.	Genotype	Species	Ploidy	Source	Accession numbers
(1)	Menggusang	*M. mongolica* Schneid.	2*n* = 2*x* = 28	Shandong, China	KF886262
(2)	Shansang	*M. bombycis* Kiodz.	2*n* = 2*x* = 28	Shandong, China	KF886263
(3)	Beiqu No.1	*M. atropurpurea* Roxb.	2*n* = 2*x* = 28	Guangdong, China	KF886264
(4)	Nongsang No.12	*M. multicaulis* Perr.	2*n* = 2*x* = 28	Jiangsu, China	KF886265
(5)	Tongxiangqing	*M. multicaulis* Perr.	2*n* = 2*x* = 28	Zhejiang,China	KF886261
(6)	Husang No.32	*M. multicaulis* Perr.	2*n* = 2*x* = 28	Jiangsu, China	KF886266
(7)	Xinyizhilai	*M. alba* L.	2*n* = 2*x* = 28	Japan	KF886267
(8)	Fu No.1	*M. alba* L.	2*n* = 2*x* = 28	Jiangsu, China	KF886268

### DNA extraction

Total DNA was extracted from fully expanded fresh leaves, using the cetyltrimethylammonium bromide (CTAB) method with minor modifications.

### Polymerase chain reaction–random fragment length polymorphism (PCR–RFLP) analysis

Ten sets of chloroplast primers were tested to amplify cpDNA non-coding regions in *Morus* spp. The list of primer sequences and PCR amplification conditions are shown in Table 2.[10,19,20] PCR amplifications were performed in a total volume of 20 μL: 25 ng of template DNA, 1× buffer, 2.0 mmol/L Mg^2+^, 0.25 mmol/L of deoxynucleoside triphosphates (dNTPs), 0.2 mol/L of each primer and 1.5 U of *Taq* polymerase (TaKaRa Co. Ltd., Japan). The amplifications were carried out using 1 cycle of 4 min at 94 °C, 35 cycles of 1 min at 94 °C, 45 s at 44–46 °C, 1–3 min at 72 °C and a 12 min final extension step at 72 °C. Ten microliters of the PCR products were examined in 1.5% agarose, stained by ethidium bromide and photographed under ultraviolet (UV) light to score the bands.

**Table 2.** Sequence and amplification conditions of cpDNA universal primer pairs used in this study.

No.	Amplified region	Forward primer (5′–3′)	Reverse primer (5′−3′)	Annealing temperature (°C)	Size (bp)	Reference
(1)	*trn*H-*trn*K	acgggaattgaacccgcgca	ccgactagttccgggttcga	55	1650	[19]
(2)	*trn*S-*trnf*M	gagagagagggattcgaacc	cataaccttgaggtcacggg	55	1650	[19]
(3)	*rbc*L	tgtcaccaaaaacagagact	ttccatacttcacaagcagc	55	1700	[10]
(4)	*trnk*1-*trnk*2	gggttgcccgggactcgaac	caacggtagagtactcggctttta	61.5	2500	[19]
(5)	*trn*C-*trn*D	ccagttcaaatctgggtgtc	gggattgtagttcaattggt	58	2950	[19]
(6)	*trn*D-*trn*T	accaattgaactacaatccc	ctaccactgagttaaaaggg	55	1200	[19]
(7)	*trn*M-*rbc*L	tgctttcatacggcgggact	gctttagtctctgtttgtgg	58	2850	[19]
(8)	*trn*F-*trn*Vr	ctcgtgtcaccagttcaaat	ccgagaaggtctacggttcg	58	2200	[20]
(9)	*trn*S-*trn*T	cgagggttcgaatccctctc	agagcatcgcatttgtaatg	55	1500	[19]
(10)	*psb*C-*trn*S	ggtcgtgaccaagaaaccac	ggttcgaatccctctctctc	55	1600	[19]

Seven restriction enzymes, *Alu*I, *Hae*III, *Hinf*I, *Hin6*I, *Rsa*I, *Mva*I and *Taq*I, were used for the digestion of the PCR-amplified cpDNA. Digestions were carried out with 3 U of each restriction endonuclease, and incubated for 5 h at 37 °C (*Alu*I, *Hae*III, *Hinf*I, *Hin6*I, *Rsa*I, *Mva*I) or 65 °C (*Taq*I). Restriction fragments were separated in 2% agarose in Tris-borate-EDTA buffer (1×), then stained by ethidium bromide and visualized under UV light to score the bands. A DL2000 ladder (TaKaRa Co. Ltd., Japan) was used as a molecular size marker.

### TrnD-trnT region sequencing

The PCR fragments of the *trn*D-*trn*T region in eight genotypes were recovered by Agarose gel extraction kit (TaKaRa, Japan). They were then purified and sequenced by Shanghai Biological Engineering Company Ltd.

### Data analysis

A binary matrix reflecting the presence (1) or absence (0) of each band was generated for each genotype. Different types were divided into two groups according to the restriction fragments polymorphism. Subsequently, eight *trn*D-*trn*T gene fragment sequences were obtained after sequencing, while the corresponding sequence of *M. indian* registered in GeneBank was used as an out-group.

The obtained cpDNAs were aligned using the Clustal X programme.[21] Mutations, such as base change, insertion or deletion of aligned sequences derived from mulberry cultivars were identified. A phylogenetic tree was constructed by the neighbour-joining method, using MAGE2 (Molecular Evolutionary Genetics Analysis; [22]).

## Results and discussion

### CpDNA PCR-RFLP

Ten corresponding cpDNA regions were successfully amplified in all mulberry genotypes examined in the present study. In this study, fragments of approximately 17.1 kb were amplified. It was found that each primer pair generated a single monomorphic fragment and there was no obvious polymorphism in size among the eight genotypes, except for *trn*D-*trn*T, which indicated that the sequence of chloroplast genome was conserved.

**Figure 1:**
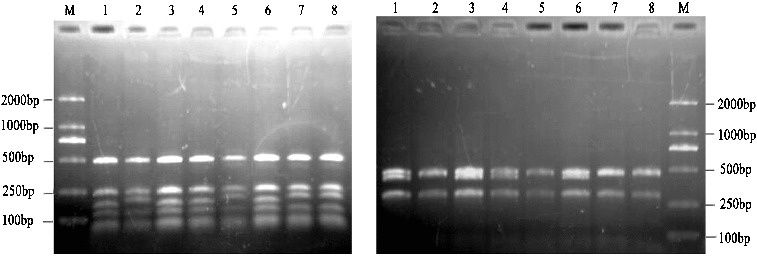
Restriction patterns of amplified and digested products of primer–enzyme combination *trn*D-*trn*T/*Hinf*I (A) and *trn*D-*trn*T /*Rsa*I (B) of chloroplast DNA from eight *Morus* L. genotypes resolved in a 2% agarose gel. Numbers were listed in Table 1. M indicates DL2000 DNA Ladder Marker.

In the present study, only 4 (5.7%) out of 70 primer–enzyme combinations were detected interspecies and intra-species polymorphism in the chloroplast genomes. A total of 152 fragments were generated by digestion of the amplified products with *Alu*I, *Hae*III, *Hinf*I, *Hin6*I, *Rsa*I, *Mva*I and *Taq*I, of which 8 (5.3%) fragments showed polymorphism. Within all regions surveyed, part of the amplified products were not digested by restriction enzymes; for example, in *trn*H-*trn*K, *rbc*L and *psb*C-*trn*S the corresponding restriction sites of *Mva*I, *Hin6*I and *Taq*I are not detected, respectively. Of the 10 restriction regions surveyed, only *trn*D-*trn*T digestion was able to generate polymorphism (Figure 1(A) and 1(B)). The genetic information generated by RFLPs was clearly shown in the combinations of *trn*D-*trn*T primers and *Alu*I, *Hinf*I, *Mva*I and *Rsa*I enzymes. Interspecies polymorphism was revealed in the digestion patterns, which indicated that base change had taken place in the sequence of cpDNA in the course of evolution. On the other hand, variations in the band length were visible by digesting the amplified products, for instance 225 bp in *trn*D-*trn*/*Alu*I (Figure 1(A)) and 470 bp in *trn*D-*trn*/*Rsa*I (Figure 1(B)). These results revealed that the insertions or deletions had occurred in the chloroplast genome.

### CpDNA genetic diversity in mulberry

Based on the patterns of *trn*D-*trn*T region digestion by four restriction enzymes, eight genotypes could be divided into two groups: Group I – ‘Menggusang’ (*M. mongolica*); ‘Beiqu No.1’ (*M. atropurpurea*); ‘Nongsang No.12’ (*M. multicaulis*); ‘Husang No.32’ (*M. multicaulis*); and Group II – ‘Shansang’ (*M. bombycis*), ‘Tongxiangqing’ (*M. multicaulis*); ‘Xinyizhilai’ (*M. alba*); ‘Fu No.1’ (*M. alba*).

Both clusters indicated that these sequences were homologous, and their female parent had the same genotype or close genetic relationships. In the genotypes of *M. multicaulis*, the ‘Tongxiangqing’ amplified bands were present as 225 bp (Figure 1(A)) and 470 bp (Figure 1(B)) fragments, which showed different patterns from that of ‘Nongsang No.12’ and ‘Husang No.32’. The result suggested that the germplasms in *M. multicaulis* had different progenitors. In the present study, no length polymorphisms were observed among ‘Tongxiangqing’, ‘Xinyizhilai’ (*M. alba*) and ‘Fu No.1’ (*M. alba*) by PCR-RFLP markers, despite of the clear discrepancy in their morphological characterizations, e.g. stigma and leaf shape. This result is consistent with the genetic relationships of the three genotypes based on isozyme analysis.[23] The conclusion showed it was difficult to further understand the evolution system and phylogenetic relationships fully in genus *Morus* based on the morphological characteristics. Mulberry plants originated in China and were widely distributed in different regions due to a long history of cultivation. The morphological and biological characteristics were influenced by the ecological environment. On the other hand, the fact that mulberry can be propagated asexually and sexually, and can be easily hybridized naturally and artificially, has made the genetic background of *Morus* rather complex. That is why, classification conclusions based on genetic information and morphological characteristics may often not coincide.

The genotype ‘Nongsang No. 12’, the hybrid offspring of ‘Beiqu No.1’ ×’Tongxiangqing’, had the same band types as its female parent ‘Beiqu No.1’, but differed from its male parent ‘Tongxiangqing’. ‘Nongsang No. 12’ and ‘Beiqu No.1’ had an identical sequence in the *trn*D-*trn*T region, while a 6 bp long deletion (in the 451–456 bp region) occurred and obviously differed from the genotype of the male parent and from those of the other samples. The results showed that the chloroplast genome is maternal inheritance in mulberry plants. The tested genotypes ‘Xinyizhilai’ and ‘Fu No.1’ had the same female parents but were obtained by different breeding methods. Both of them had homological sequence, in addition to one base insertion, which confirmed the genetic relationships and accordance with maternal inheritance laws in cpDNA of *Morus* L.

### *Trn*D-*trn*T sequence alignment

The lengths of the *trn*D-*trn*T in the eight genotypes ranged from 1198 bp (e.g. ‘Beiqu No.1’ and ‘Nongsang No.12’) to 1228 bp (e.g. ‘Tongxiangqing’ and ‘Xinyizhilai’). The obtained representative *trn*D-*trn*T sequences were submitted in GeneBank. Accession numbers were from KF886261-68, as showed in Table 1. The aligned length referred to ‘Tongxiangqing’ sequence; the content of A, T, C and G was 29.9%, 32.3.84%, 19.6% and 18.3%, respectively. The sequencing results showed high sequence homology to 97.8% or 100%. The alignment with the corresponding sequence of *M. indian* registered in GeneBank showed that there were six base variants (16 bp; 43 bp; 157 bp; 248 bp; 866 bp; 1115 bp) and two deletion sites (451–456 bp; 840–863 bp ) in the *trn*D-*trn*T region of accessions surveyed. ‘Menggusang’ bases (157 bp) for T and ‘Shansang’ bases (43 bp) for T had different characteristic sites. ‘Menggusang’, ‘Beiqu No.1’ and ‘Nongsang No.12’ had the same bases in 1115 bp, which was different from those in the other materials. At the 451–456 bp site, there were six base deletions in ‘Beiqu No.1’ and ‘Nongsang No.12’; in the 840–863 bp site, 23 nucleotides were deleted in ‘Menggusang’, ‘Beiqu No.1’, ‘Nongsang No.12’ and ‘Husang No.32’. ‘Beiqu No.1’ had an identical sequence with ‘Nongsang No.12’ in the *trn*D-*trn*T region.

**Figure 2:**
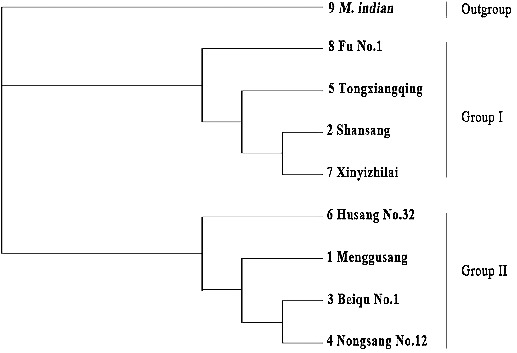
Phylogenetic tree of eight mulberry genotypes and related species *M. indian* based on the mutation sites in cpDNA *trn*D-*trn*T regions, using neighbour-joining method (MAGE).

According to the base variations and deletion site data in the *trn*D-*trn*T region, a phylogenetic tree was constructed by the neighbour-joining method (Figure 2). The tree diagram showed that the eight mulberry genotypes in this study were divided into two groups, with *M. indian* as an out-group clustering separately. ‘Shansang’ (*M. bombycis*), ‘Xinyizhilai’ (*M. alba*) and ‘Fu No.1’ (*M. alba*) were formed group I, while ‘Mongusang’ (*M. mongolica*) and ‘Beiqu No.1’ (*M. atropurpurea*) made up group II, and two groups, respectively, in different branches. Among the materials from the *M. multicaulis* species, ‘Tongxiangqing’ was clustered into group I and was completely separated from the other cultivars, ‘Nongsang No.12’ and ‘Husang No.32’, which showed that they had different original parents. The results from the DNA sequence alignment and clustering analysis suggested that the two groups of materials have the same origin of the female parent or close phylogenetic relationships, respectively.

Yang et al. [17] used PCR to amplify the chloroplast 16S ribosomal gene and digested by restriction enzymes, but obtained patterns of *Morus* materials without polymorphism. Zhao et al. [18] detected the *trn*L-*trn*F region sequence in mulberry cultivars ‘Sanglian’ and ‘Yu71-1’, and both sequences shared 95.9% homology and differed in a single base mutation. These previous results gave limited information because of the few regions and materials surveyed. Ravi et al. [24] calculated the chloroplast genome to be a total of 158484 bp in length in *M. indian*. Chen et al. [15] surveyed the sequence of *trn*L-F and *rps*16 in 67 mulberry genotypes, but the information sites for *Morus* were limited. All samples showed identical sequences in the gene-coding region of tRNA-Leu and intron. Nepal et al. [16] constructed phylogenetic trees based on separate data sets using the sequence of ITS and *trn*L-*trn*F, but they were not statistically congruent, which indicates that the 13 *Morus* species were of non-monophyletic origin. The sequence variations of the other intergenic regions in the chloroplast genome could provide genetic information for phylogenetic analyses as well, as reported in *Prunus serotina* [25] and *Elymus caninus* [26] etc. In this study, 10 cpDNA non-coding regions, accounting for 17.1 kb (about 10.79% of the chloroplast genome) were specifically amplified and 70 primer–enzyme combinations were used in an attempt to detect interspecies and intra-species polymorphism. The alignment of the *trn*D*-trn*T sequence revealed the presence of nucleotide variations and deletions of 6 bp and 23 bp in length. Further research will be carried out with a larger number of samples to provide more informative sites. The genetic variation information and the maternal inheritance mode in chloroplast genomes would provide reliable theoretical basis for the genus *Morus* system classification and phylogenetic relationship identification.

## Conclusions

In this study, 10 cpDNA primer pairs could be used for successful amplification in 8 mulberry (*Morus* spp.) genotypes, with approximately 17.1 kb of the chloroplast genome analysed. The 152 marker loci were detected by 70 primer/restriction endonuclease combinations, among which the *trn*D-*trn*T non-coding region was detected by visible fragment length variation in different cultivars. Eight *Morus* L. genotypes were divided into two groups. The *M. multicaulis* genotypes displayed diversity on an intraspecies level. The sequencing results showed that ‘Nongsang No.12’ was identical with the female parent ‘Beiqu No.1’ in the surveyed sequence, but different from the male parent ‘Tongxiangqing’, suggesting that the cpDNA was maternal inheritance in *Morus* L. There were two deletion fragments (451–456 bp; 840–863 bp) and six base point mutations in the *trn*D-*trn*T region of the tested materials. The sequence of *trn*D-*trn*T in the cpDNA of mulberry could provide more genetic information for phylogenetic analysis and pedigree identification. 
